# The Impact of the Microbiological Vaginal Swab on the Reproductive Outcome in Infertile Women

**DOI:** 10.3390/life13061251

**Published:** 2023-05-25

**Authors:** Sebastian Findeklee, Lena Urban, Romina-Marina Sima, Simona Lucia Baus, Alexander Halfmann, Gudrun Wagenpfeil, Erich-Franz Solomayer, Bashar Haj Hamoud

**Affiliations:** 1 Department of Obstetrics and Gynecology, Faculty of Medicine, Saarland University Hospital, 66424 Homburg, Germany; sebastian.findeklee@uks.eu (S.F.); lena.urban00@web.de (L.U.); simona.baus@uks.eu (S.L.B.); erich.solomayer@uks.eu (E.-F.S.); bashar.hajhamoud@uks.eu (B.H.H.); 2 Department of Obstetrics and Gynecology, UMF Carol Davila Bucharest, Bucur Maternity, 050474 Bucharest, Romania; 3 Department of Medical Microbiology and Hygiene, Faculty of Medicine, Saarland University Hospital, 66424 Homburg, Germany; alexander.halfmann@uks.eu; 4 Institute of Medical Biometry, Epidemiology and Medical Informatics, Saarland University Hospital, 66424 Homburg, Germany; gudrun.wagenpfeil@uks.eu

**Keywords:** vaginal microbiome, vaginal dysbiosis, vaginal swab, fertility treatment, endometriosis

## Abstract

Background: The thesis on which this paper is based intended to investigate whether the result of the microbiological vaginal swab has an influence on the outcome of the fertility treatment. Methods: The microbiological vaginal swabs of patients who received fertility treatment at Saarland University Hospital were evaluated. Depending on the microorganisms detected, the swab result was classified as inconspicuous, intermediate, or conspicuous. The SPSS software was used to determine the correlation between the swab result and the outcome of the fertility treatment. Results: Dysbiosis was associated with a worse outcome of fertility treatment. The pregnancy rate with a conspicuous swab was 8.6%, whereas it was 13.4% with an inconspicuous swab. However, this association was not statistically significant. Furthermore, an association of endometriosis with dysbiosis was found. Endometriosis was more frequent with a conspicuous swab result than with an inconspicuous result (21.1% vs. 17.7%), yet the correlation was not statistically significant. However, the absence of lactobacilli was significantly associated with endometriosis (*p* = 0.021). The association between endometriosis and a lower pregnancy rate was also statistically significant (*p* = 0.006). Conclusion: The microbiological vaginal and cervical swabs can be used as predictors for the success of fertility treatments. Further studies are needed to assess the impact of transforming a dysbiotic flora into a eubiotic environment on the success of fertility treatments.

## 1. Introduction

The average age of women at the birth of their first child has increased significantly in Germany since 1980 and is now 30.2 years [1]. This trend contributes to the ever-increasing importance of the use of fertility treatments. Due to scientific progress, which makes it possible to identify different microbiota, the vaginal microbiome is gaining increasing scientific attention. Its importance in the context of fertility treatment is also becoming a subject of scientific discussion. Of course, the success of fertility treatment does not depend solely on the patient’s vaginal colonization. Nevertheless, there is increasing evidence that a certain vaginal colonization is associated with a higher pregnancy rate [2]. On the other hand, dysbiosis leads to a lower pregnancy rate after fertility treatment [3,4,5]. The term dysbiosis refers to both the presence of pathogenic or facultative pathogenic microorganisms and the absence of microorganisms that contribute to the physiological vaginal flora, such as lactobacilli. By influencing vaginal dysbiosis with the help of a medical treatment, new approaches are emerging in the treatment of infertility. The aim of the underlying study was to investigate the influence of the vaginal colonization, identified using the microbiological vaginal and cervical swab, on the success of fertility treatment [6]. In the majority of present studies, the vaginal microbiota was identified with the help of 16S-RNA genome analysis. However, this method of microbiome diagnostics has mainly been used in the context of clinical studies so far and does not represent the standard in everyday clinical practice. Therefore, this study investigates whether the methods of cultural cultivation, involving the nucleic acid amplification test using PCR for microorganisms that are difficult to cultivate, also provide an indication of a beneficial or nonbeneficial vaginal flora regarding the pregnancy rate.

## 2. Materials and Methods

**Figure 1:**
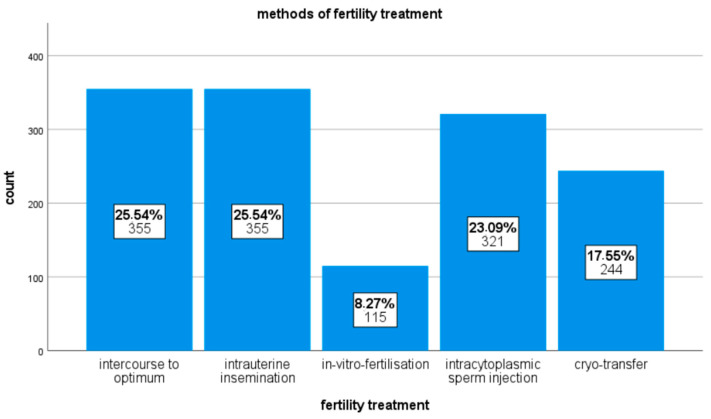
Bar chart showing the proportions of the different fertility treatments.

The data of 397 patients who were treated at the Clinic for Gynecology, Obstetrics, and Reproductive Medicine at Saarland University Hospital between January 2018 and August 2022 were evaluated retrospectively. A total of 1390 fertility treatments were recorded. The reproductive medicine procedures used were intercourse to optimum, intrauterine insemination (IUI), in vitro fertilization (IVF), intracytoplasmic sperm injection (ICSI), and the transfer of cryopreserved embryos previously obtained via one of the procedures just mentioned. The percentage shares of the different methods of fertility treatments are shown in Figure 1.

The vaginal and cervical swabs were classified as inconspicuous, intermediate, or conspicuous depending on the quality and quantity of the microorganisms detected and assigned to the type of fertility treatment carried out in each case. Table 1 shows the classification of the different types of detection into the respective category of the swab result.

The microorganisms from the vaginal samples were detected by cultural cultivation, while the cervical swabs were analyzed by polymerase chain reaction (PCR). The reason for using PCR as the detection method was the difficulty in detecting these bacteria (*Chlamydia trachomatis*, Mycoplasmataceae) by culture. In addition, the Nugent score of the vaginal swabs was determined by microscopy. If a swab result required treatment, the type of therapy carried out was documented in order to check the therapy’s influence on the outcome of the fertility treatment. The data were analyzed using the statistical program SPSS 23.0.

## 3. Results

**Figure 2:**
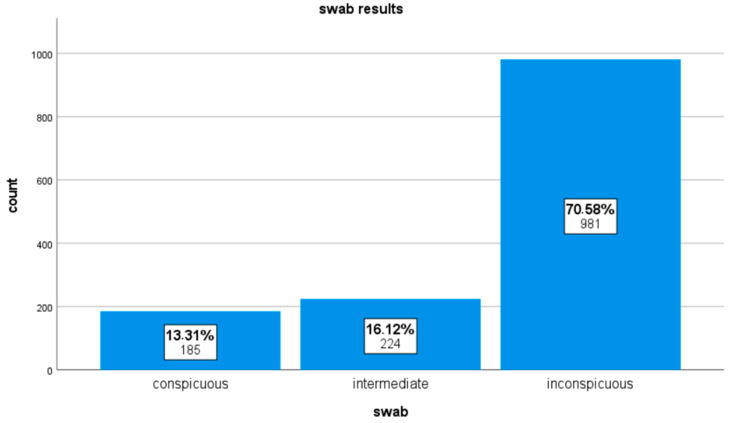
Bar chart showing the proportions of the different swab categories.

The pregnancy rate among the total of 1390 fertility treatments carried out was 13.1%. The birth rate resulting from the evaluation of the patient files was 6.3%. However, it must be noted that, in 18.1% of the pregnancies that occurred, there was no further documentation of the course of the pregnancy, as these women did not give birth at Saarland University Hospital. Therefore, the actual birth rate was most probably slightly higher than the 6.3% just mentioned. A total of 448 swabs from 397 patients were analyzed. Vaginal and cervical swabs were counted as one swab, since they were taken at the same time, and the classification as inconspicuous, intermediate, or conspicuous was made by looking at the two swab results together. In 70.6% of the fertility treatments, the swab was inconspicuous in advance, in 16.1%, the result of the swab was classified as intermediate, and, in 13.3%, it was conspicuous. These results are illustrated in the bar chart in Figure 2.

Using the model of the generalized estimating equations (GEEs), no statistically significant correlation was found between the swab result and the pregnancy or birth rate. Nevertheless, a clear difference was apparent in the group of conspicuous swab results. At 8.6%, the pregnancy rate was almost 5% lower for the conspicuous swab result, compared to the pregnancy rate of 13.4% for the inconspicuous swab result. The pregnancy rates of the different swab results are illustrated in Table 2. 

The birth rate of the group with conspicuous swabs (4.3%) was also lower than the birth rate of the group with inconspicuous swabs (6.6%). The highest pregnancy rate of 15.6% was achieved in the group with the swabs classified as intermediate. At 7.3%, the birth rate of this group was also the highest. The absence of lactobacilli was one of the criteria on the basis of which a swab was classified as conspicuous, and it was detected in the vaginal swab in advance of 10.1% of fertility treatments. The absence of this physiological flora was associated with a lower pregnancy rate. While only 7.9% of fertility treatments in which no lactobacilli could be detected in the previous swab resulted in pregnancy, the pregnancy rate with confirmed detection of lactobacilli was 13.7%. However, with a *p*-value of 0.063, this association was just above the defined significance level at which statistical significance can be assumed. 

### 3.1. Possible Confounding Variables

The average age of patients at the time they first came to the fertility clinic was comparable in all groups. In the overall collective, the average age was 32.9 years. The average BMI was slightly higher in the group of patients with inconspicuous swabs (27.32 kg/m²) than in the group of patients with conspicuous and intermediate swabs (25.96 kg/m² and 25.95 kg/m²). A significant confounder was the presence of endometriosis. Endometriosis was highly significantly (*p* = 0.006) associated with a lower pregnancy rate, as depicted in Table 3.

In addition, endometriosis was associated with dysbiosis, yet not statistically significantly. Endometriosis was simultaneously present in 24.3% of the fertility treatments among the conspicuous swab group, which was more than twice as high compared to the intermediate swab group (10.3%). In 19.6% of the fertility treatments with an inconspicuous swab result, the patient was known to have endometriosis.

### 3.2. Treatment of the Vaginal Microbiological Colonization

In 11.8% of the fertility treatments, treatment of the vaginal microbial composition was undertaken before the start of the fertility treatment based on the swab result. The most common form of treating the swab results was antibiotic treatment which was applied in advance of 5.8% of the fertility treatments. In the group of conspicuous swabs, the pregnancy rate was twice as high if antibiotics were applied compared to not applying antibiotics (14.0% vs. 7.0%). Similarly, in the group with an intermediate swab, the pregnancy rate was higher with antibiotic treatment (60.0%) than without antibiotic treatment (14.6%). Due to the small number of fertility treatments in the group with intermediate swabs in which antibiotic therapy was applied in advance, this pregnancy rate of 60% is not meaningful. The absolute and relative pregnancy rates with antibiotic treatment are shown in Table 4, Table 5 and Table 6, depending on the swab result. 

Only the pregnancy rate of the group with an inconspicuous swab, which was treated with antibiotics despite being classified as inconspicuous, was lower with antibiotic treatment (9.1%) than without antibiotic treatment (13.5%). A correlation between treatment with Fluomizin^®^, an antiseptic containing the active ingredient dequalinium chloride, and the success of the fertility treatments could not be established. In the case of probiotic treatment, the case numbers were too small to be able to make a meaningful statement. Antifungal treatment was associated with a higher pregnancy rate only if the swab was conspicuous (14.3% vs. 8.2%); however, this result was not statistically significant.

## 4. Discussion

In this study, the pregnancy rate with dysbiosis was lower than that with a eubiotic vaginal environment. The influence of certain microbiota on the success of fertility treatments was also described by Fu et al. [7]. Ventolini et al. also found a correlation between dysbiosis and poorer pregnancy outcomes from conception rate to delivery [8]. Patel et al. came to a contrary conclusion in their clinical study, in which they compared the intestinal and vaginal microbiota of infertile women with those of fertile women [9]. They concluded that the gut microbiota had very little influence on the vaginal microbiota. Surprisingly, vaginal eubiosis was more common in the group of infertile women, while vaginal dysbiosis was more common in the group of fertile women. These results show that further research is needed in this sector.

On closer examination of the abnormalities in the swab result, it became apparent that the absence of lactobacilli in particular was associated with a lower pregnancy rate, even though this association was not statistically significant. A significant correlation between the loss of lactobacilli and a lower pregnancy rate after embryo transfer was found by Tsai et al. in their clinical study [10]. The fact that there is a correlation between a lactobacillus-dominated vaginal microbiome and a higher pregnancy rate has been confirmed by numerous studies [2,5,11,12]. A fundamental question is whether the vaginal swab is at all suitable for predicting implantation success since the actual implantation takes place in the endometrium. For a long time, it was assumed that the uterus had a sterile environment. However, this assumption was already refuted in 2017 by Chen et al. whose study analyzed the microbiota of the female reproductive tract of 110 women [13]. Samples were taken from the vagina, cervix, endometrium, tubes, and peritoneal fluid. Chen et al. identified a microbiota continuum of the female reproductive tract and could, therefore, prove that the endometrium also represents a nonsterile environment. Furthermore, they provided evidence that the investigation of vaginal and cervical microbiota can be used to draw conclusions about the composition of the upper reproductive tract. In their prospective observational study, Moreno et al. showed that endometrial dysbiosis is associated with poorer reproductive outcomes in patients undergoing assisted reproductive treatments [14]. These results emphasize the possibility of using endometrial microbial composition as a biomarker to predict the success of reproductive medical treatment. Ichiyama et al. compared the vaginal with the endometrial microbiota of women with repeated implantation failure (RIF) [11]. Concordant to the study by Chen et al., this study also showed a higher alpha-diversity of the endometrium compared to the vaginal composition. In addition, the patients in whom vaginal dysbiosis was detected also had a dysbiotic uterine environment at the same time. It is remarkable that, in this study, although a significantly lower rate of lactobacilli was detected in the vaginal swab of the RIF patients, the rate of lactobacilli in the endometrial samples did not differ from that of the control group. Consequently, this study showed the superiority of the vaginal swab over the endometrial sample as a biomarker for repeated implantation failure. The prospective cohort study by Lozano et al. also compared the endometrial microbiome of a group of patients diagnosed with RIF with that of a control group without RIF [15]. Here, in contrast to the study by Ichiyama et al., a significantly lower rate of lactobacilli was found in the patients with RIF. The alpha diversity between the two groups did not differ. The heterogeneity of the current literature is underlined by the study by Kitaya et al. [16]. A higher alpha-diversity was found in endometrial fluid compared to vaginal secretions. This was observed both in patients with RIF and in the control group. In this study, only the endometrial microbiota showed significant differences in composition when comparing the RIF group with the control group. The vaginal composition of the two groups did not differ significantly. Endometrial colonization by lactobacilli was also investigated by Kyono et al. in their pilot study [17]. They compared the pregnancy rates of IVF patients with a lactobacillus-dominated microbiota (LDM) with those with a non-lactobacillus-dominated microbiota (NLDM). The pregnancy rates per embryo transfer were higher in the LDM group compared to the NLDM patients, but the difference was not statistically significant. No statistically significant difference could be found regarding the miscarriage rate either. In conclusion, colonization of the endometrium with lactobacilli, in contrast to vaginal colonization with lactobacilli, is not a distinct predictor for the success of fertility treatment.

According to current scientific knowledge, endometriosis is clearly associated with a lower pregnancy rate [18]. This thesis confirmed a statistically significant association between endometriosis and lower pregnancy rates. In addition, endometriosis was associated with dysbiosis; however, this association did not reach statistical significance. That the microbiome may be related to the development of endometriosis is still controversial in the scientific community, yet there is now growing evidence that a link may exists [19,20]. Ata et al. found a complete absence of *Atopobium* in the vaginal and cervical microbiota of the endometriosis group in their prospective observational cohort study in which they compared the microbiota of healthy women with those who had stage 3 and 4 endometriosis [21]. In addition, microorganisms containing potentially pathogenic species were elevated in the endometriosis patients. Salliss et al. concluded that there is no clear consensus on the relationship between the composition of specific microbiota and endometriosis [22]. However, they also found that bacteria associated with bacterial vaginosis and the absence of lactobacilli in the cervicovaginal microbiome were linked to the presence of endometriosis and infertility.

It is still uncertain whether dysbiosis promotes the development of endometriosis or whether endometriosis leads to dysbiosis [19,21]. An animal study by Yuan et al. showed that the Firmicutes/Bacteroidetes ratio of mice injected intraperitoneally with endometrial tissue was increased compared to the control mice [23]. However, these results refer to the gut microbiota of the mice. Further studies are needed to clarify the direction of the relationship between endometriosis and dysbiosis.

A crucial question for clinical practice is now whether the treatment of an identified dysbiosis also leads to better outcomes of fertility treatments. In the underlying study, fertility treatments with a conspicuous swab result and subsequent antibiotic therapy showed a higher pregnancy rate compared to fertility treatments with a conspicuous swab result without antibiotic treatment. However, it was also shown that antibiotic treatment had a negative effect on the pregnancy rate if the swab was inconspicuous. Studies on antibiotic therapy to treat dysbiosis so far only exist in the case of simultaneous presence of another pathology such as chronic endometritis, since the sole detection of single microorganisms that do not belong to the physiological vaginal flora would not justify antibiotic therapy. In chronic endometritis, antibiotic treatment is considered an adequate form of therapy, which also leads to improved results when methods of assisted reproduction are used [24,25]. The common characteristics of chronic endometritis and endometriosis, such as their immunological, inflammatory, and infectious aspects, studied by Kitaya et al. provide evidence for a possible benefit of antibiotic therapy in endometriosis regarding an improved live birth rate after embryo transfer [26]. Cela et al. described the association of dysbiosis, the resulting inflammatory response, and a consequent poorer IVF outcome [12]. In addition, this study investigated the effects of combined treatment with antibiotics and probiotics on dysbiosis. Although this kind of treatment resulted in pregnancy in some of the patients, the number of patients in the sample was too small to conclude that this treatment strategy had a reliable effect on the pregnancy rates. 

Prophylactic antibiotic treatment in infertile patients should be avoided as no significant benefit on reproductive outcomes can be demonstrated [27]. Eskew et al. studied the effects of prophylactic treatment with azithromycin in IVF patients [28]. During the fertility treatment, a total of three vaginal swabs were taken. The sample collection times were at the beginning of the fertility treatment (baseline), at ovule retrieval, and at the time of embryo transfer. No specific microbial composition could be associated with azithromycin treatment or pregnancy rate in any of the three samples. Therefore, treatment with azithromycin had no significant effect on bacterial composition and, thus, should be avoided because of the negative effects of antibiotic therapy such as the risk of developing resistance toward antibiotics.

If, in addition to the detection of potentially pathogenic species, a non-lactobacillus-dominant microbiome is also present, the combination of an antibiotic with a probiotic vaginal suppository is useful [29]. In general, the effect of probiotics consists of their antioxidant and immunomodulatory effect [30]. In addition, they can inhibit the formation of biofilms of pathogenic species. The patients in the underlying study were partly treated with a vaginally administered probiotic containing the strain *Lactobacillus acidophilus*. However, due to the very small number of fertility treatments in which probiotics were administered in advance, it is hardly possible to make a meaningful statement about the influence of administering probiotics on the outcome of fertility treatment. The current literature on the effect of probiotics is also heterogeneous. In their meta-analysis, López-Moreno et al. investigated the potential of vaginal probiotics in the treatment of dysbiosis [31]. They concluded that, with the help of vaginal probiotics, the relative frequency of unphysiological vaginal microbiota decreased, while an increase in the various *Lactobacillus* species was observed. The question whether a short-term administration of probiotics also leads to a long-term vaginal colonization of lactobacilli was addressed by Tomusiak et al. [32]. Their study was able to show a significant decrease in the Nugent score and the vaginal pH value, as well as a significant increase in the number of lactobacilli. In contrast to these results, the study by Jepsen et al. could not prove the effectiveness of probiotics in improving an unfavorable microbiota profile [33]. Decisive for clinical practice is not only the treatment of dysbiosis, but also whether a higher pregnancy rate can be achieved by this. Corbett et al. addressed this question in their review, in which they examined the pregnancy success of infertile couples after probiotic therapy [34]. However, the results of the studies examined were controversial in that no consensus could be found.

A limitation of this study is the non-standardized timing of the swab collection before the start of the first cycle of fertility treatment. The swabs were not always taken in a standardized way in the cycle directly before the start of the fertility treatment. In some cases, the sample was taken 7 months before the start of the actual fertility treatment and, in other cases, 2 months before the start of such treatment. Due to the retrospective design of this study, which is another limitation, it was not possible to influence the timing of sampling. However, the time of sampling was standardized insofar as it was always taken on the 12th day of the menstrual cycle. The high dynamics of vaginal colonization [33] in combination with the inconsistent timing of sample collection leads to a reduced comparability of the direct effect of a swab result on the success of fertility treatment. In order to create better comparability between different patients, future studies with a prospective design should standardize the timing of sample collection. Ultimately, it is necessary to emphasize that the unfulfilled desire to have children is complex, and there are numerous interacting factors. The success of fertility treatment does not depend solely on the vaginal or endometrial microbiome.

## 5. Conclusions

The underlying study shows that vaginal dysbiosis, particularly the absence of lactobacilli, is associated with lower pregnancy rates. Added to that, the presence of endometriosis is also associated with dysbiosis and infertility. By assessing the vaginal microbial composition and detecting bacterial species associated with poor reproductive outcomes before starting fertility treatment, it is possible to treat the dysbiosis first. This could increase the success rate of fertility treatments and reduce the number of fertility treatments that would have had a higher risk of not being successful. Nevertheless, the indication for antibiotic treatment should always be carefully considered, as the negative effects of such treatment in the presence of the physiological vaginal flora outweigh the benefits. Future studies should investigate the extent to which transformation of a dysbiotic environment into eubiosis affects the outcome of fertility treatments. The patients included in the study were not selected by specific criteria except the presence of infertility. Therefore, the results of this study can be applied to women with infertility, but not to all women.

## Figures and Tables

**Table 1:** Classification of swab results based on the quality and quantity of detected microorganisms.

Microorganism	Inconspicuous	Swab Result Intermediate	Conspicuous
*Lactobacilli*	Detection	-	No detection
*E. coli*	Sporadic/numerous	1× massive	Massive (after control)
*Klebsiella*	Sporadic/numerous	1× massive	Massive (after control)
*Bacillus cereus*	Sporadic/numerous	1× massive	Massive (after control)
*Morganella morganii*	Sporadic/numerous	1× massive	Massive (after control)
*Enterococcus species*	Sporadic/numerous/massive	-	-
Gram-negative rod bacteria	Sporadic/numerous	1× massive	Massive (after control)
Gram-negative mixed flora	Sporadic/numerous	1× massive	Massive (after control)
*Streptococcus agalactiae*	Sporadic/numerous/massive	-	-
*Staphylococcus aureus*	Sporadic/numerous/massive	-	-
Coagulase-negative staphylococci	Sporadic/numerous/massive	-	-
*Kytococcus schroeteri*	Sporadic/numerous/massive	-	-
*Pseudomonas putida*	-	-	Sporadic/numerous/massive
*Pseudomonas aeruginosa*	-	-	Sporadic/numerous/massive
*Candida species*	Sporadic/numerous	1× massive	Massive (after control)
*Chlamydia trachomatis*	-	-	PCR detection
*Mycoplasma genitalium*	-	-	PCR detection
*Mycoplasma hominis*	-	-	PCR detection
*Ureaplasma parvum*	-	-	PCR detection
*Ureaplasma urealyticum*	-	-	PCR detection
*Trichomonas vaginalis*	-	-	PCR detection

**Table 2:** Correlation between swab result and pregnancy rate.

			Pregnancy		Total
			Yes	No	
Swab	Conspicuous	Count	16	169	185
		% within swab	8.6%	91.4%	100.0%
	Intermediate	Count	35	189	224
		% within swab	15.6%	84.4%	100.0%
	Inconspicuous	Count	131	850	981
		% within swab	13.4%	86.6%	100.0%
Total		Count	182	1208	1390
		% within swab	13.1%	86.9%	100.0%

**Table 3:** Correlation between swab result and endometriosis.

			Endometriosis		Total
			Yes	No	
Swab	Conspicuous	Count	12	45	57
		% within swab	21.1%	78.9%	100.0%
	Intermediate	Count	8	72	80
		% within swab	10.0%	90.0%	100.0%
	Inconspicuous	Count	55	256	311
		% within swab	17.1%	82.3%	100.0%
Total		Count	75	373	448
		% within swab	16.7%	83.3%	100.0%

**Table 4:** Correlation between antibiotic treatment and pregnancy rate in case of conspicuous swab result.

			Pregnancy		Total
			Yes	No	
Antibiotic	Yes	Count	6	37	43
		% within antibiotic	14.1%	86.0%	100.0%
	No	Count	10	132	142
		% within antibiotic	7.0%	93.0%	100.0%
Total		Count	16	169	185
		% within antibiotic	8.6%	91.4%	100.0%

Swab = conspicuous.

**Table 5:** Correlation between antibiotic treatment and pregnancy rate in case of intermediate swab result.

			Pregnancy		Total
			Yes	No	
Antibiotic	Yes	Count	3	2	5
		% within antibiotic	60.0%	40.0%	100.0%
	No	Count	32	187	219
		% within antibiotic	14.6%	85.4%	100.0%
Total		Count	35	189	224
		% within antibiotic	15.6%	84.4%	100.0%

Swab = intermediate.

**Table 6:** Correlation between antibiotic treatment and pregnancy rate in case of inconspicuous swab result.

			Pregnancy		Total
			Yes	No	
Antibiotic	Yes	Count	3	30	33
		% within antibiotic	9.1%	90.9%	100.0%
	No	Count	128	820	219
		% within antibiotic	13.5%	86.5%	100.0%
Total		Count	131	850	981
		% within antibiotic	13.4%	86.6%	100.0%

Swab = inconspicuous.

## Data Availability

Third-party data were used in this study.
